# Assessing the routine-practice gap for home blood pressure monitoring among Chinese adults with hypertension

**DOI:** 10.1186/s12889-020-09901-0

**Published:** 2020-11-23

**Authors:** Hui-Juan Zuo, Ji-Xiang Ma, Jin-Wen Wang, Xiao-Rong Chen

**Affiliations:** 1 Department of community health research, Beijing Anzhen Hospital, Capital Medical University, Beijing Institute of Heart Lung and Blood Vessel Diseases, No.2 Road Anzhenli, Chaoyang District, Beijing, 100029 China; 2 Department of Chronic Non-communicable Diseases Prevention, Chinese Center for Disease Control and Prevention, Beijing, 100050 China

**Keywords:** Hypertension, Home blood pressure monitoring, Blood pressure control, Office blood pressure

## Abstract

**Background:**

Home blood pressure monitoring (HBPM) is recommended for diagnosis, treatment adjustment and management of most hypertension cases in hypertension guidelines from multiple countries. This study aimed to evaluate HBPM behaviour and explore the routine-practice gap in HBPM among Chinese adults with hypertension.

**Methods:**

Data were collected from 20 communities across three cities and six townships in three provinces (Beijing, Shandong and Jiangsu) in China between October 2014 and November 2014. In total, 2272 patients with hypertension aged ≥35 years that were registered with a primary health station in their local communities were selected by simple random sampling.

**Results:**

Among the 2272 participants, 45.3% owned a home blood pressure (BP) monitor. In addition, 27.5% (625/2272) engaged in HBPM weekly or more frequently. Healthcare providers’ advice was the strongest factor contributing to home BP monitor ownership and weekly HBPM behaviour, with odds ratios of 13.50 and 8.97, respectively. Approximately 4.4% of participants had achieved optimal HBPM regimens (duplicate measurements in the morning and evening for 7 days). Patients with uncontrolled office-measured BP were more likely to conduct HBPM regularly in the morning and evening, measure their BP two or three times in each session and maintain 7 consecutive days of HBPM than patients with controlled office BP (8.8% vs. 5.8%, *P* = 0.042; 14.3% vs. 8.1%, *P* = 0.002; and 19.9% vs. 12.4%, *P* = 0.005, respectively). Only 16.0% (165/1030) of participants actively reported their HBPM readings to doctors.

**Conclusion:**

The HBPM strategies specified in hypertension guidelines are seldom achieved in actual practice in China. Only a small proportion of patients actively participate in using HBPM to enhance their hypertension care. HBPM may be improved by healthcare providers offering specific advice and training.

**Supplementary Information:**

The online version contains supplementary material available at 10.1186/s12889-020-09901-0.

## Background

Previous studies have shown that home blood pressure monitoring (HBPM) is a useful tool to improve long-term adherence to antihypertensive drug treatment among patients with hypertension, and thereby improve hypertension control rates [1–4]. HBPM is also more efficacious for predicating morbidity and mortality associated with cardiovascular disease (CVD) than blood pressure (BP) measurement conducted in a healthcare provider’s office [5–7]. Hypertension guidelines from multiple countries [8–10] emphasize the importance of HBPM for diagnosis, treatment adjustment and long-term follow-up for most hypertension cases, and recommended incorporating HBPM in clinical practice. Following recommended best practice can facilitate the successful implementation of HBPM and impact how hypertension is managed in primary care settings [11, 12].

A HBPM protocol commonly specified in recent hypertension guidelines [8–10] involves morning and evening BP measurements. Ideally, this should comprise two to three readings (at least two readings) at each measurement session taken for 7 consecutive days before a clinic visit. There are minor differences in content regarding BP monitoring in available guidelines. For example, the 2018 European Society of Cardiology (ESC)/European Society of Hypertension (ESH) guidelines for hypertension [8] recommend two measurements on each occasion over a minimum of 3 days, with a preferred period of 6–7 days. The 2018 Chinese guideline [9] and 2017 American College of Cardiology (ACC)/American Heart Association (AHA) hypertension guidelines [10] recommend two to three readings at each measurement session for 7 consecutive days. The present study was conducted in 2014, and our simulation of ideal HBPM regimens was based on recommendations that were consistent across the 2013 ESC/ESH hypertension guidelines [13], 2008 AHA/ASH hypertension guidelines [14] and 2012 Chinese expert consensus on family blood pressure monitoring (i.e. monitoring protocol consistent with the 2018 China hypertension guidelines [9]). These guidelines recommended weekly HBPM (once in the morning and once in the evening) for controlled hypertension and more frequent HBPM for uncontrolled hypertension. Optimally, patients should perform HBPM every morning and evening, and take two to three measurements each time for 7 consecutive days before a clinic visit.

Currently, most healthcare providers encourage patients with hypertension to monitor their own BP at home [11, 12]. Previous studies reported proportions of patients with hypertension ranging from 29.7 to 84.0%, and noted that patients indicated their doctor encouraged them to perform HBPM [15–18]. However, the prevalence of HBPM (at least once a week) in patients with hypertension varies, being lower than 30% in some countries and over 50% in others [15, 18]. An optimal HBPM schedule (i.e. duplicate measurements in the morning and evening for 7 days) has not yet been reported in a population-based study. In addition, little is known about the use of HBPM data to inform treatment adjustment. Therefore, we conducted this study to clarify home BP monitor ownership, regular HBPM behaviour and the application of HBPM readings for treatment adjustment. We also explored the factors making the strongest contributions to HBPM improvement to minimise the gap between guidelines and actual HBPM practice.

## Methods

This cross-sectional survey was conducted in October–November 2014. The study area included three cities (Qingdao city, Wuxi city and Beijing) and six townships (Rushan, Yiyuan, Lianshui, Sheyang, Gaobeidian and Fatou) located in Shandong Province, Jiangsu Province and Beijing. Patients with hypertension who were registered with primary health stations in their local communities were selected to participate in this study. Inclusion criteria were: 1) aged over 35 years; and 2) a medical history of hypertension for a minimum of 3 months. Exclusion criteria were: 1) patients suffering from serious congestive heart failure, renal insufficiency and requiring dialysis treatment, and patients with cancer; and 2) patients registered in the community health records who did not live in that community. The required sample size was estimated by: *n* = [t_α_2P (1-P)]/d^2^, α = 0.05, t_a_ ≈ 2, d = 0.1P. Among patients with hypertension in China, ownership of a home BP monitor ranges from 35 to 65% in different areas. Therefore, a sample size of 750 patients (350 patients from urban areas and 350 patients from rural areas) was considered sufficient for each province. In total, 20 communities were selected: two urban communities and six rural communities from Jiangsu and Shandong province, respectively, and two urban communities and two rural communities from Beijing. Each selected community provided community health records for all patients with hypertension who met the inclusion criteria. Participants were randomly selected from this database using SPSS for Windows version 18.0, based on proportional values. In addition, 15% of the entire sample size was also randomly selected from the community health records for replacement in cases of response failure. In total, 2272 participants were recruited. Written informed consent was obtained from participants, each of whom was required to complete a face-to-face interview using an interviewer-led questionnaire.

A structured questionnaire was developed by a team of researchers at the Beijing Institute of Heart Lung and Blood Vessel Diseases and the Chinese Center for Disease Control and Prevention. The details of the questionnaire are shown in Additional File 1 (Table S1, Table S2 and Table S3). The survey questionnaire was pilot-tested with 30 volunteers with hypertension in the community of Jiangsu to determine participants’ level of comprehension. The results of the pilot study were not included in this paper.

The questionnaire covered demographic information, family and medical history, lifestyle, antihypertensive medications, Home BP monitor ownership and measurement behaviour, and physical examination. Ownership of a home BP monitor was evaluated with one item: ‘Do you have an upper-arm automatic electronic monitor in your home?’ The item assessing HBPM behaviour comprised four questions: ‘How many days did you conduct HBPM per week?’; ‘What time did you measure your blood pressure each day?’; ‘How many times did you conduct in each measuring session?’; and ‘Did you perform HBPM for 7 consecutive days during the last 3 months?’ Application of HBPM readings was evaluated with two questions that assessed if participants’ doctors requested their HBPM results and whether they proactively reported their HBPM results to their doctors. Participants’ height, weight and BP were measured during a physical exam. Office BP was measured using Omron HBP-7071 with a standard cuff size (a bladder 12 cm wide and 22–26 cm long for most patients, and larger cuffs for obese patients or those with an arm circumference > 32 cm) on the right arm, supported at the level of the heart. BP was measured with the participant in a sitting position at fixed time from 9:00 to 12:00 in the morning, after taking antihypertensive medicines. The participants rested for at least 5 min in a seated position, and two BP measurements were taken 1–2 min apart and averaged for the record. An additional measurement was taken if the first two readings differed by > 5 mmHg, and the mean value of the three readings was recorded in these cases [9, 10]. The home BP values were collected by means of questionnaires completed by participants. They were asked to provide the last home BP values in the past week (If they didn’t measure home BP in the past week, home BP readings within 14 days should be provided). We intended to assess home BP control at any time, in the morning and evening using the mean value of two readings, respectively. But 96.2% of participants filled in only one BP value on the questionnaire, which was used for home BP assessment directly. The rest of participants filled in both morning and evening BP values. Therefore, mean value of BP was calculated for home BP assessment.

We defined uncontrolled office BP as systolic BP (SBP) ≥140 mmHg and/or diastolic BP (DBP) ≥90 mmHg, and controlled office BP as SBP < 140 mmHg and DBP < 90 mmHg. Optimal home BP control was defined as a BP < 135/85 mmHg. Home BP monitor ownership was defined as participants who had an upper-arm automated electronic BP monitor in their home. The prevalence of HBPM was defined as the proportion of participants who measured their BP at home and conducted measurement at least weekly. Regular HBPM behaviour was defined as participants who: 1) conducted HBPM at least weekly; 2) took at least two readings 1–2 min apart in the morning before taking medications and in the evening before supper; and 3) conducted HBPM for 7 consecutive days before a clinic visit. History of ischemic stroke was defined as: a history of symptoms/signs such as numbness, abnormal speech, transient blindness, vertigo, nausea, deviated eyes and mouth, hemiplegia or dribbling; consistent signs on a brain computed tomography or magnetic resonance imaging; and a diagnosis of ischemic stroke by a neurologist (including cerebral thrombosis or lacunar infarction). The presence of coronary heart disease (CHD) was defined by a history of coronary artery bypass grafting or coronary stent implantation, or hospitalisation for myocardial infarction. A family history of ischemic CVD was defined as a diagnosis of ischemic stroke or CHD in a first-degree relative (sibling or parent). Current smokers were defined as those who reported having smoked ≥100 cigarettes during their lifetime and were smoking every day or some days at the time of interview. Current alcohol drinkers were defined as drinking alcohol once or more per week during the previous 12 months. For adult males, alcohol drinking was defined as daily drinking over 750 ml of beer, over 250 ml of wine, or over 75 g of 38-degree alcoholic liquor. For adult females, these quantities were 450 ml, 150 ml and 50 g, respectively. Body mass index (BMI) was calculated as body mass in kilograms divided by height in meters squared. An overweight status was defined as a BMI of 24.0–27.9 kg/m^2^, and obesity was defined as a BMI of ≥28.0 kg/m^2^.

Data were input twice to guarantee data accuracy. If the data from the two databases were inconsistent, the original data on the questionnaire were rechecked. Statistical analyses were performed with SPSS for Windows version 18.0 (IBM Corp., Armonk, NY, USA). Chi-square tests were used to compare differences in proportions of different groups. Multiple backward stepwise logistic regression analysis was performed to identify factors influencing home BP monitor ownership and weekly HBPM behaviour. On the basis of chi-square tests, significant variables were entered into a multivariate model. Odds ratios (OR) and corresponding 95% confidence intervals (CI) were calculated for each independent variable. All reported *P*-values were two-tailed, and *P*-values less than 0.05 were considered statistically significant.

## Results

In total, 2295 participants were enrolled in this study. After eliminating 23 incomplete questionnaires 2272 participants (male *n* = 977, female *n* = 1295) were included in the analyses. Participants’ age ranged from 35 to 93 years, with a mean age of 64.2 ± 10.9 years. In total, 1141 (50.2%) participants were from urban areas and 1131 (49.8%) were from rural areas. Statistically significant differences were identified for all characteristics across the three provinces (Beijing, Jiangsu and Shandong) except sex. Table 1 presents participants’ demographic characteristics, separated by city.

**Table 1:** General characteristics of study participants, *n* (%)

Characteristics	Total subjects	Different area			
		Beijing (*N* = 761)	Jiangsu (*N* = 758)	Shandong (*N* = 753)	*P*-Value
**Sex**					0.089
Male	977 (43.0)	344 (45.2)	333 (43.9)	300 (39.8)	
Female	1295 (57.0)	417 (54.8)	425 (56.1)	453 (60.2)	
**Age group (y)**					0.002
< 65	1091 (48.0)	337 (44.3)	402 (53.0)	352 (46.7)	
≥ 65	1181 (52.0)	424 (55.7)	356 (47.0)	401 (53.3)	
**Location of living**					
Urban	1141 (50.2)	383 (50.3)	380 (49.8)	378 (50.2)	0.944
Rural	1131 (49.8)	378 (49.7)	378 (50.2)	375 (49.8)	
**Educational level**					< 0.001
Intermediate school or lower	1761 (77.5)	506 (66.5)	550 (72.6)	705 (93.6)	
Middle and High school	359 (15.8)	175 (23.0)	142 (18.7)	42 (5.6)	
≥ College graduate	152 (6.7)	80 (10.5)	66 (8.7)	6 (0.8)	
**Duration of hypertension (y)**					< 0.001
< 5	829 (36.5)	226 (29.7)	269 (35.5)	334 (44.4)	
5–9	673 (29.6)	251 (33.0)	194 (25.6)	228 (30.3)	
≥ 10	770 (33.9)	284 (37.3)	295 (38.9)	191 (25.4)	
**Family history and medical history**					
Family history of ISCVD	390 (17.2)	173 (22.7)	125 (16.5)	92 (12.2)	< 0.001
History of ISCVD	240 (10.6)	118 (15.5)	69 (9.1)	53 (7.0)	< 0.001
History of diabetes	458 (20.2)	190 (25.0)	142 (18.7)	126 (16.7)	< 0.001
**Lifestyle**					
Current smoking	378 (16.6)	89 (11.7)	162 (21.4)	127 (16.9)	< 0.001
Current drinking	412 (18.1)	111 (14.6)	168 (22.2)	133 (17.7)	0.001
Overweight or obesity	1516 (66.7)	386 (50.7)	518 (68.3)	612 (81.3)	< 0.001

*ISCVD* Ischemic cardiovascular disease, *HBPM* Home blood pressure monitoring

In total, 1598 (70.3%) participants were treated for hypertension, 991 (46.3%) had controlled office BP and 1541 (67.8%) had received advice on HBPM from a healthcare professional. There were 1030 (45.3%) participants who had a home BP monitor: 77.1% in Beijing, 34.1% in Jiangsu and 23.9% in Shandong. HBPM was performed weekly or more frequently by 27.5% (625/2272) of participants. Table 2 shows home BP monitor ownership and prevalence of HBPM by participants’ characteristics. Home BP monitor ownership was likely to be higher in patients who were older, had a higher level of education, were treated with antihypertensive drugs, had controlled hypertension, had a history of CVD, lived in urban locations and had received advice regarding HBPM from a healthcare professional. The prevalence of HBPM was higher in patients who were aged 65 years and over, treated with antihypertensive drugs, lived in urban locations and received advice from a healthcare professional.

**Table 2:** Proportion of home BP monitor ownership and prevalence of HBPM, *n* (%)

	All subjects *n* = 2272	Owning a home BP monitor		Conducting HBPM weekly	
		Yes (%)	*P*-value	Yes (%)	*P*-value
Sex			0.104		0.123
Male	977	462 (47.3)		285 (29.2)	
Female	1295	568 (43.9)		340 (26.3)	
Age (y)			< 0.001		< 0.001
< 65	1091	375 (34.4)		212 (19.4)	
≥65	1181	655 (55.5)		413 (35.5)	
Area			< 0.001		< 0.001
Beijing	761	587 (77.1)		364 (47.8)	
Jiangsu	758	263 (34.7)		150 (19.8)	
Shandong	753	180 (23.9)		111 (14.7)	
Location of living			< 0.001		0.012
Urban	1141	703 (61.6)		445 (39.3)	
Rural	1131	327 (28.9)		180 (15.8)	
Educational level			< 0.001		< 0.001
Intermediate school or lower	1761	618 (35.1)		380 (21.6)	
Middle and High school	359	290 (80.8)		168 (46.8)	
≥College graduate	152	122 (80.3)		77 (50.7)	
History of ISCVD			< 0.001		0.004
Yes	240	135 (56.3)		85 (35.4)	
No	2032	895 (44.0)		540 (26.6)	
Duration of hypertension (y)			< 0.001		< 0.001
<5	829	282 (34.0)		140 (16.9)	
5–9	673	317 (47.1)		194 (28.8)	
≥10	770	431 (56.0)		291 (37.8)	
Antihypertensive Medication			< 0.001		< 0.001
Yes	1598	804 (50.3)		507 (31.7)	
No	674	226 (33.5)		118 (17.5)	
Controlled hypertension			0.004		0.241
Yes	991	483 (48.7)		285 (28.8)	
No	1281	547 (42.7)		340 (26.5)	
Advice on HBPM from doctor			< 0.001		< 0.001
Yes	1541	952 (61.8)		584 (37.9)	
No	731	78 (10.7)		41 (5.6)	

*ISCVD* Ischemic cardiovascular disease, *HBPM* Home blood pressure monitoring

Multivariate regression analysis showed that age, urban versus rural location, education level, other family members with hypertension, history of CVD, antihypertensive medication, office BP control and advice on HBPM from healthcare providers were associated with home BP monitor ownership. All of the above factors were correlated with weekly HBPM behaviour, except history of CVD. Healthcare providers’ advice was the strongest factor contributing to home BP monitor ownership and weekly HBPM behaviour, with ORs of 13.50 and 8.97, respectively (Table 3).

**Table 3:** Multivariate analysis on factors influencing home BP monitor ownership and weekly HBPM behaviour

Factors	Owning a home BP monitor			Conducting HBPM weekly		
	OR	95% CI	*P*-value	OR	95% CI	*P*-value
Age group	1.44	1.18–1.77	< 0.001	1.82	1.46–2.25	< 0.001
Location of living	0.30	0.28–0.33	< 0.001	0.29	0.26–0.31	< 0.001
Education level	2.04	1.85–2.26	< 0.001	1.58	1.44–1.74	< 0.001
Duration of hypertension	1.47	1.26–1.71	< 0.001	1.67	1.28–1.99	< 0.001
Other family members with hypertension	1.62	1.23–2.14	0.001	1.46	1.13–1.89	0.004
History of ISCVD	1.52	1.08–2.14	0.016	–	–	–
Antihypertensive medication	1.75	1.39–2.18	< 0.001	1.21	1.06–1.40	0.006
Advice on HBPM from doctor	13.50	10.24–17.79	< 0.001	8.97	6.40–12.57	< 0.001

*ISCVD* Ischemic cardiovascular disease, *HBPM* Home blood pressure monitoring

Sex, BP control was adjusted in the analysis on factors influencing home BP monitor ownership

Sex, BP control, history of ISCVD was adjusted in the analysis on factors influencing weekly HBPM behaviour

We also conducted logistic regression analysis for treated patients with hypertension to show the relationship between controlled office BP and HBPM (*n* = 1598). Controlled office BP was significantly associated with education level, duration of hypertension, current drinking status and BMI, with ORs of 1.35 (95% confidence interval [CI]: 1.23–1.48), 0.81 (95%CI: 0.68–0.95), 0.66 (95%CI: 0.48–0.88) and 0.61 (95%CI: 0.49–0.76), respectively. No significant relationship was found between office BP control and weekly HBPM, even after adjusting for the other covariates. Additional File 1 (Table S4) shows the estimated association between controlled office BP and other factors.

Table 4 shows participants’ regular HBPM behaviour. Of those who had a home BP monitor, the prevalence of HBPM was 59.0% in participants with controlled office BP and 62.2% in those with uncontrolled office BP. In addition, 5.8% of participants with controlled office BP conducted HBPM regularly in the morning and evening, compared with 8.8% of those with uncontrolled office BP (*P* = 0.042). Furthermore, 8.1% of participants with controlled office BP measured their BP two or three times, compared with 14.3% of those with uncontrolled office BP (*P* = 0.002). Participants with uncontrolled office BP (19.9%) were more likely to conduct HBPM for 7 consecutive days than those with controlled office BP (12.4%, *P* = 0.005). Overall, participants with controlled office BP had a lower optimal HBPM rate (duplicate measurements in the morning and evening for 7 days) than those with uncontrolled office BP (2.1% vs. 6.4%; *P* = 0.001).

**Table 4:** Regular HBPM behaviour among participants who had a home BP monitor, n (%)

Behaviour of HBPM	Controlled office BP (*n* = 483)	Uncontrolled office BP (*n* = 547)	*P*-value
**Days of HBPM per week**			0.302
Less than one day	198 (41.0)	207 (37.8)	
One or more days	285 (59.0)	340 (62.2)	
**Time of HBPM**			0.042
Irregular	376 (77.8)	390 (71.3)	
Regular in morning or evening	79 (16.4)	109 (19.9)	
Regular in morning and evening	28 (5.8)	48 (8.8)	
**Readings at each measurement session**			0.002
Only one reading	444 (91.9)	469 (85.7)	
Two or three readings	39 (8.1)	78 (14.3)	
**Consecutive seven days of HBPM**			0.005
Never	423 (87.6)	438 (80.1)	
Ever	60 (12.4)	109 (19.9)	
**Optimal HBPM schedule**	10 (2.1)	35 (6.4)	0.001

*HBPM* Home blood pressure monitoring

We asked participants who performed HBPM if their doctors requested their HBPM results and if they proactively reported their HBPM results to their doctors. Doctors requested HBPM results from 31.8% (328/1030) of participants. Doctors were more likely to request HBPM results from those with uncontrolled office BP than those with controlled office BP (7.9% vs. 53%, *P* <  0.001). Only 16.0% (165/1030) of participants proactively reported their HBPM readings to their doctors, and this was statistically significantly higher in those with uncontrolled office BP than those with controlled office BP (5.2% vs. 25.6%, *P* <  0.001).

Participants who performed HBPM were requested to provide the latest readings. Among those with controlled office BP, the control rate of home BP was 76.0% (367/483), and it was more likely to be higher among those with an office BP < 120/80 mmHg. Only a few participants provided morning (*n* = 78) or evening (before bedtime) (*n* = 57) BP values. The control rate of morning and evening BP was 52.6 and 64.9%, respectively. Optimal morning BP was higher among participants with an office BP < 120/80 mmHg, but the difference was not significant (*P* = 0.061) (Table 5).

**Table 5:** HBP control among patients with controlled office BP

Controlled office BP (mmHg)	Optimal HBP control n (%)	Optimal morning BP control n (%)	Optimal evening BP control n (%)
< 120/80 (*n* = 116)	99/116 (85.3)	17/25 (68.0)	11/15 (73.3)
≥120/80 (*n* = 367)	268/367 (73.0)	24/53 (45.3)	26/42 (61.9)
*P*-value	0.007	0.061	0.426

*HBP* Home blood pressure

## Discussion

This study assessed the status of home BP monitor ownership and regular HBPM behaviour among Chinese adults with hypertension. The results showed that nearly half of the participants had a home BP monitor, but this varied across different provinces. More than one-quarter of participants reported weekly HBPM. The HBPM regimens specified in hypertension guidelines are seldom achieved in actual practice. Only a small group of participants actively discussed their HBPM readings with their doctors. Healthcare professionals’ advice was the strongest factor contributing to home BP monitor ownership and weekly HBPM.

Use of HBPM has progressively increased over the last two decades. Initially, around 20% of patients with hypertension in developed countries used HBPM [18, 19], with current rates reported as 31–75% in studies across different countries [15–18]. A large difference was also found across different areas of China; with performing HBPM at least once a week reported by 52.0% in Zhejiang, 36.9% in Chengdu [20] and 42.8% in Beijing [21]. The present study showed that HBPM was performed by around 47.8% of patients with hypertension in Beijing, 19.8% in Jiangsu and 14.7% in Shandong. The majority of previous studies showed a higher prevalence of HBPM among urban residents; however, rural residents comprised half of our sample, and we found a low proportion of rural participants had a home BP monitor.

Various factors may affect an individual purchasing a home BP monitor and using HBPM. Previous studies among general patients with hypertension (primary care or community settings) have shown inconsistent results. Some studies reported that patients with a higher education level, higher income, male sex and a younger age were more likely to adopt HBPM [2, 15, 22, 23], whereas other studies found higher HBPM use in older adults [16, 23, 24]. In this study, no difference in HBPM was found between males and females, and older participants were more likely to use HBPM. Similarly, HBPM use has been associated with healthcare providers’ advice on HBPM [12, 16, 24]. A study among patients with chronic kidney disease revealed the most common reason for not using HBPM was lack of advice by a physician (43.4%) [25]. Another study showed that 35.2% of patients were advised to perform HBPM by their doctor, with this proportion being 29.7% in Canada [16], about 50% in Japan [26] and the UK [27] compared with 62.1% in our study. Those results suggested that healthcare professionals should promote HBPM use, especially among patients with hypertension who are younger, newly diagnosed with hypertension and live in rural areas.

Previous studies defined regular HBPM as a respondent’s self-report of monitoring their own BP at home, and performing this at least weekly [16, 23]. According to relevant hypertension guidelines [13, 14], we defined regular HBPM more clearly and completely. Performing HBPM regularly (duplicate measurements in the morning and evening for 7 days) is seldom achieved in current practice; about 4.4% of participants in our study had achieved optimal HBPM regimens. Uptake of both morning and evening measurement was low, especially in those with controlled office BP. According to the US National Health and Nutrition Examination Survey 2009–2010, patients with uncontrolled BP engaged in weekly or more frequent HBPM, whereas patients who achieved the ideal control standard engaged in monthly HBPM [28].

HBPM use remains low and poor adherence to hypertension guidelines may be attributable to barriers at the patient, clinician, and healthcare system levels [11]: 1) patients lack adequate knowledge about the optimal regimen for HBPM [20, 24, 29]; 2) presence of barriers to conducting morning and evening measurement for patients [11]; 3) lack of encouragement from healthcare providers or more detailed direction for HBPM not being provided to patients [25]; and 4) HBPM readings seldom being documented by clinicians [30]. Healthcare systems should further enhance the successful implementation of HBPM, supported by plans to encourage healthcare professionals and provide patients with HBPM regimens, such as selection of appropriate BP measurement devices, measurement conditions and self-measurement skills and protocols.

Clinicians seldom use HBPM instead of office measurements for treatment adjustment. A Canadian study noted that only 19.0% of primary physicians used HBPM readings to guide therapy [30], although about 30% of patients shared their HBPM results with their health professional [16, 30]. In the present study, 31.9% of participants were asked their HBPM results by their doctors, and only 16.0% proactively shared their readings with their doctors. With the development of BP telemonitoring technology and equipment, Internet-based remote monitoring and home management of BP is expected to further improve the application of HBPM readings [31].

Optimal BP control at home was obtained among participants with controlled office BP, but optimal morning BP control was significantly lower than other time BP control. Previous studies showed 50–60% of patients with controlled office BP had an elevated morning BP [32, 33]. Our findings are consistent with previous publications involving similar populations in that 47.4% did not achieve the target for morning BP control. Morning BP is now recognised to be superior to office BP in predicting cardiovascular risk, Therefore, hypertension guidelines highlight the assessment of morning BP [34].

Several limitations of this study should be noted. First, given that the sample size was not large, the results may not be representative of all urban and rural areas in Shandong, Jiangsu and Beijing. Second, we did not investigate patients’ knowledge of HBPM and common reasons for not using HBPM, and therefore cannot validate assumptions regarding such reasons. Third, there was no consideration of advice from healthcare providers to investigate their efficacy; even if there was standard plan for HBPM in the guideline, guideline adherence was not evaluated. Fourth, the rate of home BP control was not representative because the results were not based on the uniform measuring standard and were from a small group of patients. In addition, we did not confirm whether participants measured their home BP using appropriate BP monitor (validated or not, appropriate cuff size) and a proper measurement technique. These details might have led to measurement errors. Reporting bias also existed because home BP monitors did not have a log-memory function.

## Conclusions

In this study, we found that home BP monitor ownership was relatively low in rural areas and varied across different areas in China. The recommendations for HBPM as specified in hypertension guidelines are seldom achieved in current practice among Chinese adults with hypertension. Patients with uncontrolled hypertension are more likely than those with controlled hypertension to report their home BP readings to their doctors, but only a small proportion actively participate in using home BP measurements to enhance their care. HBPM may achieve greater success through specific advice and training by healthcare providers. New systems are needed to systematically collect home BP data and provide this information routinely to health professionals.

## Supplementary Information

**Additional file 1. **Home BP monitor ownership and measurement of a structured questionnaire developed for this study and independent determinants of controlled office BP among treated hypertensive sample. The survey instrument used in the study to assess home BP monitor and routine-practice of HBPM among Chinese adults with hypertension. **Table S1.** Home BP monitor ownership and routine practice of HBPM survey. The questions and possible responses used in the assessment of home BP monitor ownership, HBPM weekly and optimal HBP monitoring behavior. **Table S2.** Health care providers’ HBPM practice survey. The data used to assess the value of health care providers on HBPM and the application of HBPM readings for treatment adjustment. **Table S3.** Physical examination. The data used in the assessment of overweight or obesity and control of office blood pressure. **Table S4.** Summary of independent determinants of office BP control. OR and 95% CI indicated the correlation between HBPM weekly or other factors and controlled office BP among treated hypertensive patients.

## Data Availability

The raw dataset analyzed in the current study are available from the corresponding author on reasonable request.

## Abbreviations

BP: Blood pressure
HBP: Home blood pressure
HBPM: Home blood pressure monitoring
CHD: Coronary heart disease;
CVD: Cardiovascular disease
OR: Odds ratios
CI: Confidence intervals
