# Supplementary material for: Assessing the routine-practice gap for home blood pressure monitoring among Chinese adults with hypertension
Source: BMC Public Health. 2020 Nov 23;20:1770. doi: 10.1186/s12889-020-09901-0 (PMC7686714; doi:10.1186/s12889-020-09901-0)
Supplement: Supplementary file 1 — Additional file 1. Home BP monitor ownership and measurement of a structured questionnaire developed for this study and independent determinants of controlled office BP among treated hypertensive sample. The survey instrument used in the study to assess home BP monitor and routine-practice of HBPM among Chinese adults with hypertension. Table S1. Home BP monitor ownership and routine practice of HBPM survey. The questions and possible responses used in the assessment of home BP monitor ownership, HBPM weekly and optimal HBP monitoring behavior. Table S2. Health care providers’ HBPM practice survey. The data used to assess the value of health care providers on HBPM and the application of HBPM readings for treatment adjustment. Table S3. Physical examination. The data used in the assessment of overweight or obesity and control of office blood pressure. Table S4. Summary of independent determinants of office BP control. OR and 95% CI indicated the correlation between HBPM weekly or other factors and controlled office BP among treated hypertensive patients. [file 12889_2020_9901_MOESM1_ESM.zip › Table S1R3.doc]

**Table S1**. Home BP monitor ownership and routine practice of HBPM survey. The questions and possible responses used in the HBPM section of the survey

Home BP monitor ownership and routine practice of HBPM

| Item | Responses |
| --- | --- |
| 1. Do you have an upper-arm automated electronic sphygmomanometer in your home? | No  Yes |
| 2. Did you perform home blood pressure measurement | No  Yes |
| 3. How many days did you conduct HBPM per week? | <1 day/W  1-2 days/W  ≥ 3 days/W |
| 4. What time did you measure your blood pressure each day? | Morning time only  Evening time Only  Morning time and evening time  Irregular |
| 5. How many times did you conduct in each measuring session? | One time  Two or three times  More than three times |
| 6. Did you conduct HBPM for 7 consecutive days during the last three month? | No  Yes |
| 7. The last home blood pressure values in the past week. | In morning time  Reading 1: (SBP/DBP),  Reading 2: (SBP/DBP  In evening time  Reading 1: (SBP/DBP)  Reading 2: (SBP/DBP)  At all the other times  Reading 1: (SBP/DBP),  Reading 2: (SBP/DBP) |

BP: blood pressure; HBPM: home blood pressure monitoring; SBP: systolic blood pressure; DBP: diastolic blood pressure
